# The Seroprevalence of *Toxoplasma gondii* in Chinese Population With Cancer: A Systematic Review and Meta-analysis

**DOI:** 10.1097/MD.0000000000002274

**Published:** 2015-12-18

**Authors:** CaiXiao Jiang, ZhanZhan Li, Peng Chen, LiZhang Chen

**Affiliations:** From the Department of Epidemiology and Health Statistics, School of Public Health, Central South University, Changsha, Hunan Province, China (CJ, ZL, LC); and Xiangya Medical School, Central South University, Changsha, Hunan Province, China (PC).

## Abstract

Toxoplasmosis is fatal in the immunocompromised individuals such as cancer patients with chemotherapy. Clinical toxoplasmosis in cancer patients is a great public health concern in China. We performed this meta-analysis to assess the seroprevalence and odds ratios (ORs) of *Toxoplasma gondii* in Chinese population with cancer compared with those without.

A methodical literature search was conducted with the help of the PubMed, Web of Knowledge, Embase, Chinese Web of Knowledge, Wanfang, and Chongqing VIP database. Case-control studies published from their inception until April 2015, reporting the seroprevalence of *T. gondii* in Chinese population with cancer, were covered as well. The nonweighted prevalence, pooled random-effects estimates of ORs, and 95% confidence intervals (CIs) were all calculated.

Nineteen studies including 4493 cases and 6797 controls were incorporated in the meta-analysis. The overall seroprevalence of *T. gondii* was higher in population with cancer compared with those without ((20.59% vs 6.31%, *P* < 0.001; OR 3.90, 95% CI 3.00–5.07). The OR of *T. gondii* in cancer patients is further subgrouped according to publication year, sample size, and diagnostic methods. The pooled OR estimates were 4.80 (95% CI 2.57–8.99) from 1991 to 1999, 4.96 (95% CI 3.03–8.12) during 2000 to 2005, and 2.94 (95% CI 2.46–3.50) during 2006 to 2015. The pooled OR estimates were 6.16 (95% CI 3.87–9.78) when the sample size was below 400, 5.37 (95% CI 3.84–7.53) when the sample size was between 400 and 500, and 2.58 (95% CI 2.17–3.07) when the sample size was above 500. The pooled OR estimates were 5.50 (95% CI 3.98–7.62) by using indirect hemagglutination assay method, and 3.15 (95% CI 2.67–3.72) by using enzyme-linked immunosorbent assay method.

The meta-analysis study found Chinese population with cancer had higher seroprevalence rates of *T. gondii* compared with those without.

## INTRODUCTION

*Toxoplasma gondii* infects all warm-blooded vertebrates and imposes a major burden on human and animal health. Toxoplasmosis is one of the more common parasitic zoonoses with worldwide distribution caused by the protozoan parasite *T. gondii*,^1^ which infects one-third of the world's population.^2^ Humans become infected postnatally by ingesting tissue cysts from undercooked meat containing tissue cysts, ingesting food or drink contaminated with oocysts from infected cat feces and congenitally, or by accidentally ingesting from the environment.^3^*T. gondii* is transmitted to its final host through the consumption of an intermediate host, and can infect any warm-blooded vertebrate, including humans.^4^ Seroprevalence of Toxoplasma infection widely varies from around 6.1% to 74.5% among different regions of the world.^5^ In healthy humans, infection with *T. gondii* is usually asymptomatic. If *T. gondii* infects a pregnant woman with no previous exposure to the parasite, it can migrate through the placenta into the fetus and subsequently cause congenital toxoplasmosis.^5^ However, Toxoplasmosis is fatal in the immunocompromised individuals such as cancer patients with chemotherapy, HIV/AIDS patients, and organ-transplant recipients.^6^

Cancer is the leading cause of death in economically developed countries, and in developing countries it is the second leading cause of death.^7^ Deaths from cancer worldwide are projected to continue to rise, and the number of deaths will be up to 11 million deaths in 2030.^8^ China is the most populous nation in the world and the Chinese population has a high rate of cancer. In China, the numbers of new cases and deaths from cancer are increasing year by year. In 2008, it was 2.82 million (22.3% of world total) and 1.96 million (25.9%), and the number will forecast to 4.87 million and 3.60 million by 2030.^9^ Clinical toxoplasmosis in cancer patients is a great public health concern in China. Toxoplasmosis can lead to the active parasitemia and life-threatening disease due to the rupture of pre-existent cysts, contributing to worsening of the clinical condition.^10^ Hence, for this reason, a meta-analysis was carried out to assess the seroprevalence and odds ratios (ORs) of *T. gondii* in Chinese population with cancer compared with those without.

## MATERIALS AND METHODS

### Ethical Approval

The ethical approval was not necessary in the review articles.

### Strategy for Literature Search

The available literature on the seroprevalence of *T. gondii* in Chinese population with cancer were obtained by perusing through the PubMed, Web of Knowledge, Embase, Chinese Web of Knowledge, Wanfang, and Chongqing VIP database. The literature was restricted to the Chinese and/or English language, and searched from their inception until April 2015, with the search themes of “*Toxoplasma gondii* or toxoplasmosis or *T. gondii*”, combined with “cancer or oncology or malignant neoplasm or malignant tumor”.

### Criteria for Inclusion and Exclusion

Strict criteria for inclusion and exclusion were used in this meta-analysis, and all selected studies had to meet the given inclusion criteria: studies using a case-control design in mainland China; the participants of cancer group must be patients diagnosed with cancer; participants of control group must be taken from normal population without cancer; sample size of case group must be more than 30 adults with cancer; and must provide information about the seroprevalence of *T. gondii* in cancer group and control group. Similarly, only those studies were excluded that were animal studies; repeated studies; review studies or abstracts; and the studies which included only cancer patients.

Any disagreements with the selected studies were resolved by discussion and the involvement of another author.

### Data Extraction

Two investigators performed the data by using a standardized data extraction from each article. In case of a disagreement between the 2 researchers, a discussion would be carried out to arrive at an agreement. Information was extracted from each study: general information, including name of the first author, year of publication, total sample size, collection criteria of controls, number of patients with positive test results, and *T. gondii* diagnostic methods.

### Quality Assessment

The Newcastle–Ottawa Scale^11^ was used to assess the quality of included studies.

### Statistical Analysis

The association between the seroprevalence of *T. gondii* and cancer in Chinese population was estimated by means of ORs and corresponding 95% confidence intervals (CIs). The implication of the pooled OR was determined by *Z* test. The models used were the fixed-effects model or random-effects model. The model to be used was determined by whether or not there existed heterogeneity among studies.^12,13^ The stability of sensitivity was assessed by eliminating 1 single study each time.^14^ The potential of publication bias was examined by Begg funnel plot and Egger test.^15–17^ In this study, meta-analyses were conducted by using STATA version 12.0 software (Stata Corporation, College Station, TX), and *P* < 0.05 was considered to be statistically significant.

## RESULTS

### Search Result and Characteristic of Included Studies

**FIGURE 1:**
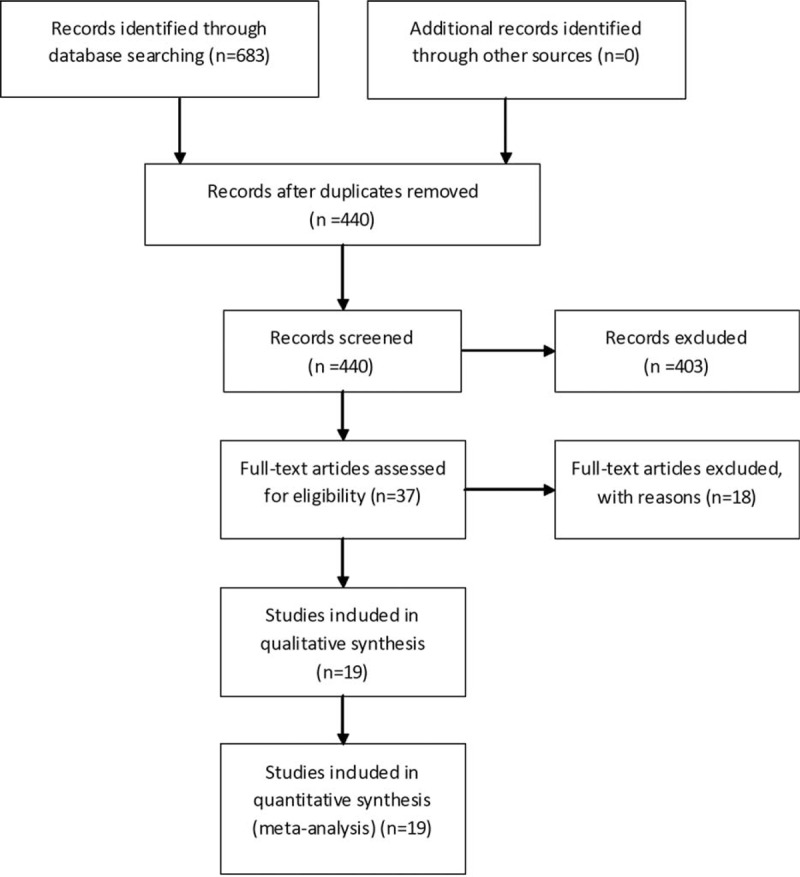
Flow diagram of included/excluded studies.

The systematic literatures search yielded 683 relevant articles. After a careful examination of each article's abstract, 440 were considered as having potential value and the full texts were retrieved for detailed evaluation. Then 403 potentially relevant articles were excluded from the meta-analysis because of their obvious irrelevance to the purpose of this study. At the end, 19 studies including 4493 cases and 6797 controls, whose dates of publication were from their inception to April 2015, were deemed eligible and were incorporated in the meta-analysis.^18–36^Figure 1 shows the flow diagram for the selection of included studies. The individuality and characteristics of these articles are given in Table 1.

**TABLE 1:**
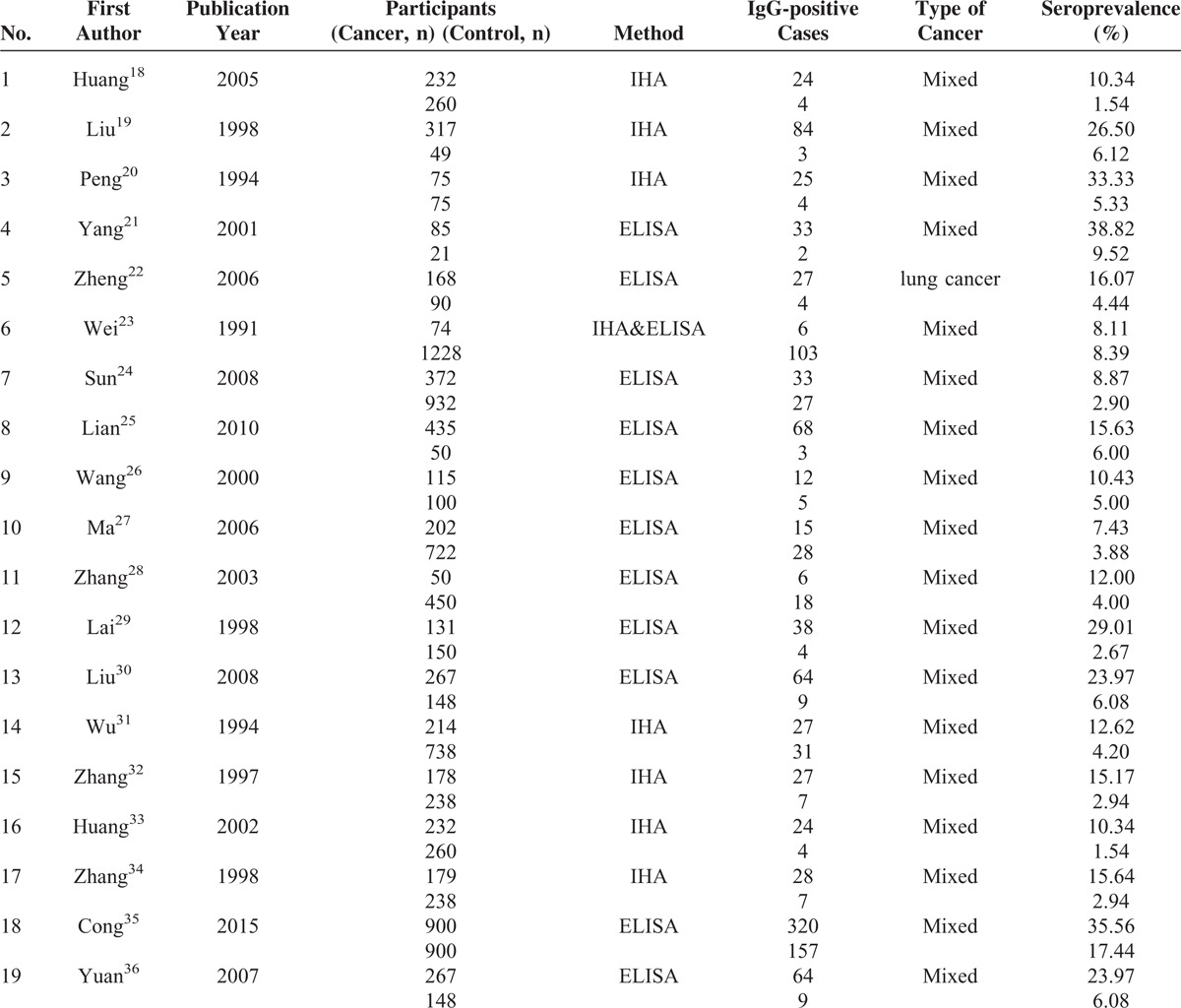
Characteristics of Included Studies

### Risk of Bias Assessment

The quality of included studies is presented in Table 2. The higher scores reflect the better study quality, and the average scores of the included studies were above 6.

**TABLE 2:**
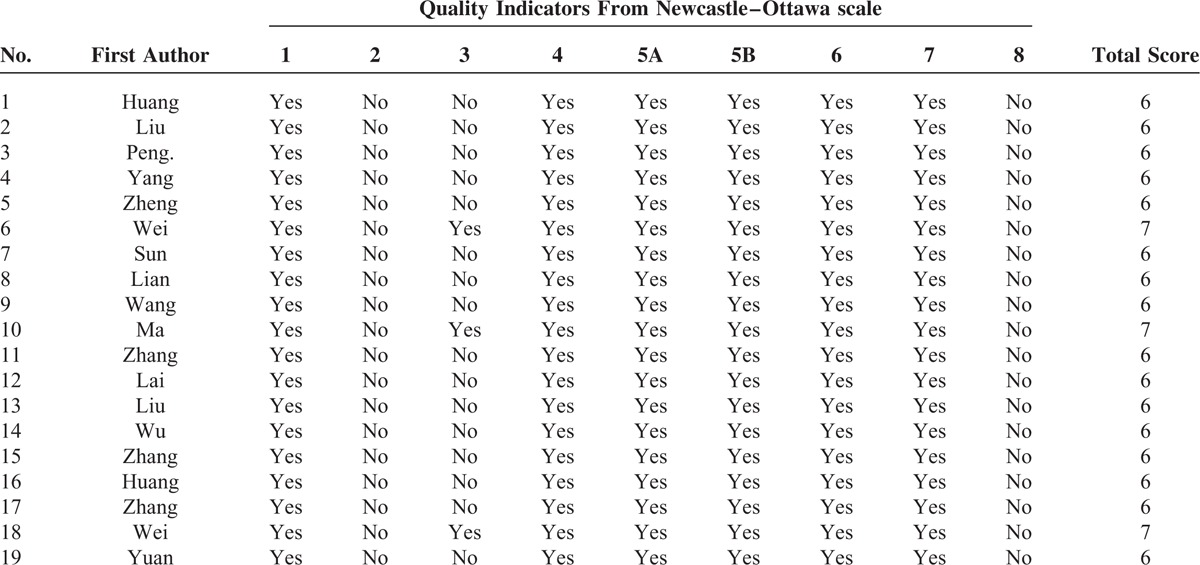
Assessment of Study Quality

### Prevalence Rates of Seroprevalence of *Toxoplasma gondii* in Cancer Patients

The overall seroprevalence of *T. gondii* was higher in a population with cancer compared with a population without (*P* < 0.001). The overall seroprevalence of *T. gondii* was 20.59% in the Chinese population with cancer, and the seroprevalence of *T. gondii* was 6.31% in the noncancer group.

### Odds Ratios of *Toxoplasma gondii* in Cancer Patients

**FIGURE 2:**
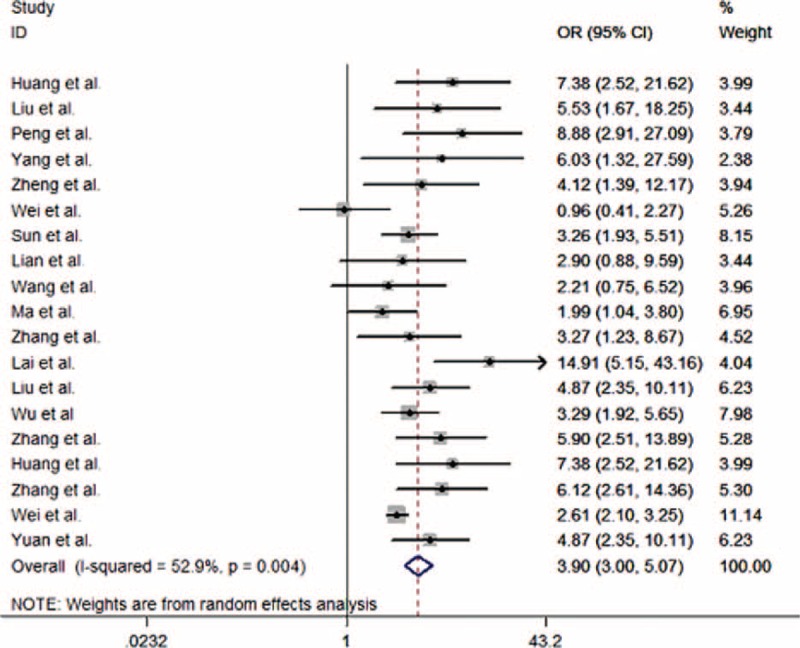
Forest plot for the meta-analysis of *Toxoplasma gondii* in Chinese population with and without cancer.

A pooled random-effects meta-analysis was conducted using data from 19 studies, which estimated the levels of *T. gondii* in Chinese population with cancer compared with that without. This analysis included data for 4493 cases with cancer and 6797 controls without cancer. As shown in Figure 2, the odds of *T. gondii* was associated with a 3.90-fold increased risk of cancer patients when compared with the control group (OR 3.90, 95% CI 3.00–5.07). However, the heterogeneity analysis of the effect sizes of depression (*Q* = 38.22, *P* = 0.004, *I*^2^ = 52.9%) showed that there was a relatively high-level heterogeneity in our meta-analysis.

### Subgroup Analyses

The OR of *T. gondii* in cancer patients was further subgrouped according to publication year, sample size, and diagnostic methods, and the OR of these subgroups is presented in Table 3. The pooled OR estimates were 4.80 (95% CI 2.57–8.99) from 1991 to 1999, 4.96 (95% CI 3.03–8.12) during 2000 to 2005, and 2.94 (95% CI 2.46–3.50) during 2006 to 2015. It was also apparent that the pooled OR estimate decreased with the increase in sample size. For instance, the pooled OR estimates were 6.16 (95% CI 3.87–9.78) when the sample size was below 400, 5.37 (95% CI 3.84–7.53) when the sample size was between 400 and 500, and 2.58 (95% CI 2.17–3.07) when the sample size was above 500. The pooled OR estimates were 5.50 (95% CI 3.98–7.62) by using indirect hemagglutination assay (IHA) method, and 3.15 (95% CI 2.67–3.72) by using enzyme-linked immunosorbent assay (ELISA) method. Information regarding the heterogeneity of the data as well as the publication bias is depicted in Table 3.

**TABLE 3:**
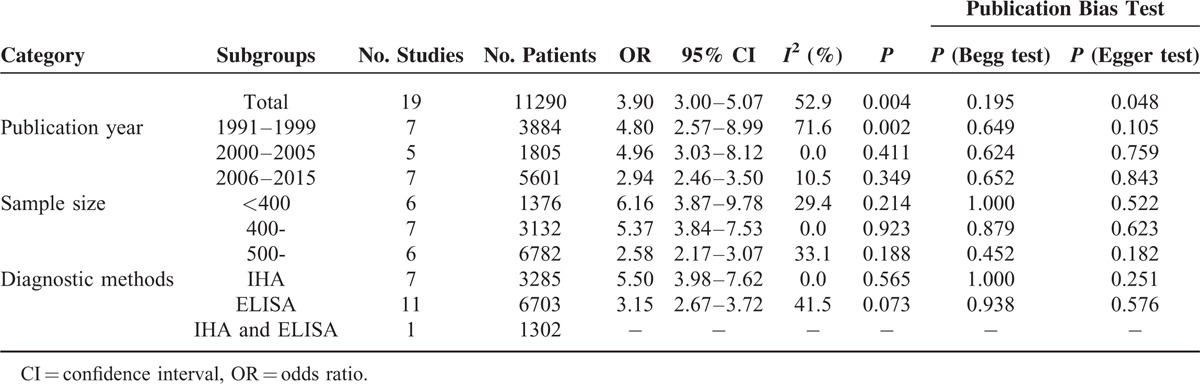
Odds Ratios of *Toxoplasma gondii* in Chinese Population With and Without Cancer (Subgroup Analyses)

### Publication Bias

**FIGURE 3:**
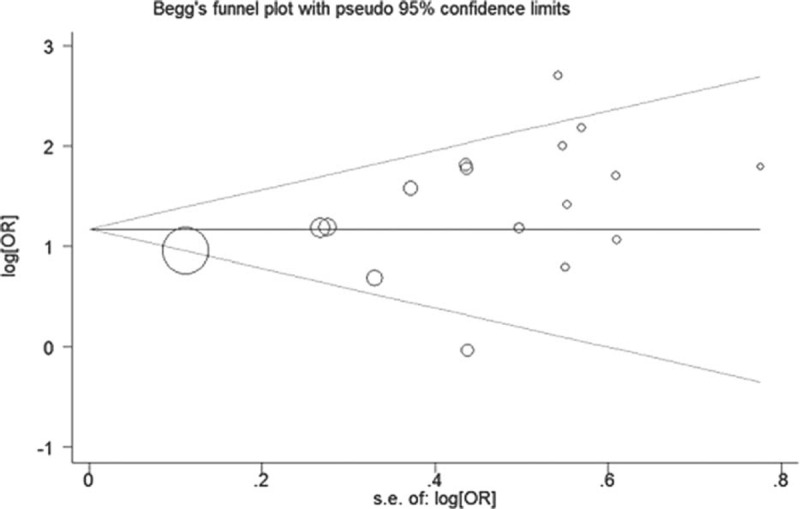
Begg funnel plot for publication bias test.

Begg test and Egger test were used to give value to the publication bias. No significant bias in the included studies (*P* = 0.195) was revealed using the modified Begg linear regression test (Figure 3). However, a significant publication bias in the included studies was observed using the Egger test (*P* = 0.048) (Table 3).

## DISCUSSION

*Toxoplasma gondii* is an extremely successful protozoal parasite, which infects almost all mammalian species including humans.^37^ Although most infections are clinically asymptomatic, in immunocompromised patients, it can cause severe disease manifestations and even death.^38^ It is a big problem that the cancer patients were seropositive for *T. gondii* tested. The cancer may induce severe opportunistic parasitic disease in antitumor treatment process. This is the first meta-analysis that aimed to assess the seroprevalence and ORs of *T. gondii* in Chinese population with cancer compared with that without. In this review, 19 studies including 4493 cases and 6797 controls were incorporated, and we found the overall seroprevalence of *T. gondii* was higher in a population with cancer compared with that without in the Chinese population. The overall seroprevalence of *T. gondii* was 20.59% in the Chinese population with cancer. The results were higher than some studies assessing the nation-wide prevalence (7.88%)^39^ and the prevalence of psychiatric patients (11.3%) in China.^40^ Meanwhile, the results were similar to this situation in foreign countries.^41–43^

This systematic review showed the level of *T. gondii* was significantly higher in cancer patients when compared with those without (OR 3.90, 95% CI 3.00–5.07). The cancer patients with low immune function were significantly more susceptible than those without.^44^

The serological tests are regarded as the gold standard and are used for diagnosis of *T. gondii* infection. The most common methods of diagnosis are SFT, indirect immunofluorescence assay, direct agglutination test, latex agglutination test, ELISA, improved self-adaptive Genetic Algorithms, and the IgG avidity test.^5^ This review revealed that IHA and ELISA techniques are the most common method used for *T. gondii* detection in China. Both humoral and cell-mediated mechanisms are included in the immunologic response to *T. gondii* infection.^45^

We must become aware of the heterogeneity that is present in our study, so subgroup analyses were done to explore potential sources of heterogeneity including publication year, sample size, and diagnostic methods. In the subgroup analyses, the pooled OR estimates were 4.80 from 1991 to 1999, 4.96 during 2000 to 2005, and 2.94 during 2006 to 2015. It was also apparent that the pooled OR estimates decreased with the increase of sample size. For instance, the pooled OR estimates were 6.16 when the sample size was below 400, 5.37 when the sample size was between 400 and 500, and 2.58 when the sample size was above 500. The pooled OR estimates were 5.50 by using IHA method and 3.15 by using ELISA method. When we grouped items according to publication year, sample size, and diagnostic methods, then the heterogeneity of different sample sizes and diagnostic methods decreased, so differences in sample size and diagnostic methods may cause this heterogeneity.

The study faces numerous possible drawbacks which may influence the result. First, we confined our studies published in English and Chinese, and this did not include the unpublished researches; in this way related unpublished studies may be left out and this method could direct to the oversight of some related studies. Second, the heterogeneity is present in our study. Third, the present meta-analysis was based on case-control studies. The case-control study is a type of observational study and it may also be more difficult to establish the timeline of exposure to disease outcome. So we could not determine the causation between the development of cancer and *T. gondii* infection. Finally, no significant bias in the included studies (*P* = 0.195) was revealed using the modified Begg linear regression test. However, a significant publication bias in the included studies was observed using the Egger test (*P* = 0.048). Apart from these drawbacks, there exist some key benefits in the analysis. This is the first meta-analysis that aimed to assess the seroprevalence and OR of *T. gondii* in the Chinese population with cancer compared with those without. Likewise, the relationship between the development of cancer and *T. gondii* infection is statistically more persuasive than any single study.

## CONCLUSIONS

The Chinese population with cancer had higher seroprevalence rates of *T. gondii* compared with those without.
